# Patient-reported anxiety and depression measures for use in Indian head and neck cancer populations: a psychometric evaluation

**DOI:** 10.1186/s41687-021-00316-y

**Published:** 2021-06-07

**Authors:** Chindhu Shunmugasundaram, Haryana M. Dhillon, Phyllis N. Butow, Puma Sundaresan, Mahati Chittem, Niveditha Akula, Surendran Veeraiah, Claudia Rutherford

**Affiliations:** 1 Faculty of Science, School of Psychology, Centre for Medical Psychology and Evidence-based Decision-making, The University of Sydney, Sydney, New South Wales Australia; 2 Faculty of Science, School of Psychology, Psycho-Oncology Cooperative Research Group, The University of Sydney, Sydney, New South Wales Australia; 3 Radiation Oncology Network, Western Sydney Local Health District, Sydney, New South Wales Australia; 4 Sydney Medical School, Faculty of Health & Medicine, The University of Sydney, Sydney, New South Wales Australia; 5 Department of Liberal Arts, Indian Institute of Technology Hyderabad, Sangareddy District, India; 6 Department of Psycho-Oncology, Cancer Institute (WIA), Chennai, India; 7 Faculty of Science, School of Psychology, Quality of Life Office, The University of Sydney, Sydney, New South Wales Australia; 8 Susan Wakil School of Nursing and Midwifery, Cancer Nursing Research Unit, The University of Sydney, Sydney, Australia

## Abstract

**Background:**

Head and neck cancers (HNC) are one of the most traumatic forms of cancer because they affect essential aspects of life such as speech, swallowing, eating and disfigurement. HNCs are common in India, with over 100,000 cases being registered each year. HNC and treatment are both associated with considerable anxiety and depression. With increasing multinational research, no suitable measures in Indian languages are available to assess anxiety and depression in Indian HNC patients. This study evaluated the psychometric properties of cross-culturally adapted versions of Zung’s self-rating Anxiety Scale (SAS) and the Patient health questionnaire – 9 (PHQ-9) in Tamil, Telugu and Hindi speaking Indian HNC populations.

**Methods:**

HNC patients were recruited from three tertiary cancer centres in India. Patients completed the cross-culturally adapted versions of SAS and PHQ-9. We assessed targeting, scaling assumptions, construct validity (exploratory and confirmatory factor analyses), convergent validity, and internal consistency reliability.

**Results:**

The study sample included 205 Tamil, 216 Telugu and 200 Hindi speaking HNC patients. Exploratory and confirmatory factor analyses indicated a two-factor solution for PHQ-9 and four-factor solution for SAS in all three languages. Cronbach’s alpha coefficients ranged between 0.717 and 0.890 for PHQ-9 and between 0.803 and 0.868 for SAS, indicating good reliability. Correlations between hypothesized scales were as expected providing evidence towards convergent validity.

**Conclusions:**

This first psychometric evaluation of the measurement properties of Tamil, Telugu and Hindi versions of the SAS and PHQ-9 in large, Indian HNC populations supported their use as severity and outcome measures across the disease and treatment continuum.

**Supplementary Information:**

The online version contains supplementary material available at 10.1186/s41687-021-00316-y.

## Background

Cancer of the head and neck (HNC) is common across the globe, with approximately 800,000 patients being registered each year [1, 2]. In developing countries such as India, due to high rates of smoking and tobacco chewing, about 200,000 people are diagnosed with HNC each year. Nearly 75% of these cases present with advanced disease and studies have found this is owing to low awareness about cancer and negative effects of tobacco and alcohol usage [3, 4]. It is estimated that oral cavity cancers account for nearly 25% of all cancer deaths in India [3]. Hence, the rates of cancer mortality and patients surviving the disease with debilitating side effects are both high. The head and neck region constitute body parts crucial for physiological functions, social interaction, communication, expression and appearance [5]. HNC treatment affects these regions and fundamental functions of life such as taste, voice, speech, breathing, hearing, eating, chewing, swallowing, facial features resulting in psychological morbidities such as low self-esteem, body image, stress, depression, anxiety and low health-related quality of life (HRQL) [6–8].

International studies have found that between 12% and 40% of all cancer patients have some form of anxiety or depressive disorder [8, 9]. However, anxiety and depression are commonly underdiagnosed and undertreated in patients with cancer [9]. This is because psychological distress to an extent is considered a normal reaction to a cancer diagnosis [10, 11]. Studies including Indian cancer populations have found similar results; about half of patients treated for HNC positively screen for anxiety or depressive disorder or both [12]. Patients with HNC are at considerably greater risk of psychological morbidity because of visible structural deformities in the facial region or breakdown in communication resulting in body image issues or emotional isolation that arise as a result of treatments such as surgery or radiation [12–14]. Treatment related morbidities and problems vary according to the type and sub-type of HNC. Certain morbidities such as disfigurement, pain, dry mouth, difficulty eating/chewing and sexual dysfunction do not recover quickly after treatment, and consequently negatively affect HRQL. For HNC patients with low self-esteem, distress levels are significant even before the start of HNC-related treatment.

In India, cancer care facilities are few, but patient burden is high, stretching already limited available healthcare resources. Hence, little focus is given to psychological morbidities and HQRL. In addition, Indian patients show reluctance to confide openly to their healthcare professionals or family members and sometimes tend to minimize the severity of symptoms they experience as a result of disease and treatment and how they affect their psychological well-being. These are some reasons for underdiagnosed psychological morbidity in India [15, 16]. This emphasizes the importance of psychological screening for all HNC patients throughout their cancer trajectory in order to identify psychological concerns such as anxiety and/or depression that might otherwise go unreported.

Patient-reported outcomes (PROs) such as anxiety and depression can be assessed with PRO measures (PROMs). PROMs are increasingly used in clinical practice to monitor patient progress and as a communication facilitator between patients and healthcare professionals [17]. Despite the benefits of using PROMs clinically, most PROMs were developed in English, making them non-usable with non-English speaking populations around the world [18, 19]. For instance, in countries like India, there are officially 22 different languages with over 700 dialects, and English is only spoken in the big cities, excluding participants from rural areas where nearly 65% of the total population reside [20]. Similarly, with changing global migration trends, English speaking countries have seen an increasing number of non-English speaking ethnic populations. Therefore, it is essential to develop or translate measures to languages other than English. This will also enable the participation of non-English speaking patients in national and multi-national clinical trials.

Our previous work identified the Zung self-rating anxiety scale (SAS) and Patient-health questionnaire 9 (PHQ-9) as HNC-relevant and psychometrically robust PROMs of anxiety and depression [18, 21–23]. Both have been culturally adapted into Tamil, Telugu and Hindi and found to be conceptually, linguistically and culturally equivalent to their original English versions [19] following methods proposed by The European Organisation for Research and Treatment of Cancer (EORTC) and Mapi Research Trust (MAPI) guidelines for translation and cultural adaptation of PROMs. This process was executed separately for both measures in each target language and involved an exhaustive process. This included translating measures (forward and back translation by two independent translators and reconciliation), reporting of problems encountered during language translation (by the translation team), their resolution (led by an expert in each language), pre-testing in the target population (10 HNC patients per language group) exploring item difficulty, clarity, relevance and sensitivity of the translation and determining equivalence in terms of content and meaning with the original. Furthermore, HNC related health professionals (three per target language) were interviewed and asked if the items were understandable, captured original concepts (while comparing both original and translated versions) and were relevant to HNC population. The Indian languages Tamil, Telugu and Hindi were chosen because these were the most commonly spoken languages in Southern and Northern parts of India, respectively. This study aimed to undertake a psychometric evaluation of the Tamil, Telugu and Hindi versions of SAS and PHQ-9.

## Methods

### Research question

To what extent are the Tamil, Telugu and Hindi versions of SAS and PHQ-9 psychometrically valid and reliable?

This study forms part of a larger program of research to translate and evaluate HNC-specific PROMs assessing body image, unmet needs, anxiety and depression into three Indian languages – Tamil, Telugu and Hindi ([18, 19], (Shunmugasundaram, C., et al: Body image scale: Evaluation of the psychometric properties in three Indian head and neck cancer language groups, in preparation), [24, 25]).

### Study setting

We conducted this study in three participating tertiary hospitals in India (Cancer Institute, WIA in Chennai, for Tamil patients, MNJ Institute of Oncology and Regional Cancer centre in Hyderabad, for Telugu patients and Nanavati Super Specialty Hospital, Mumbai for Hindi patients) between August 2019 and February 2020. Ethics approvals were obtained from The University of Sydney Human Research Ethics Committee (Sydney, Australia) [Ref: 2019/202], Scientific Advisory Committee and Ethical Committee of Cancer Institute, WIA [Ref: IEC/2019/Sep 02], Ethical Committees of MNJ Institute of Oncology & Regional Cancer Centre [Regd No: ECR/Inst/AP/2013/RR-16.) dated, 18/04/2019. No reference number provided in letter of approval] and Nanavati Super Specialty Hospital.

### Sample

Patients of either gender, with an HNC diagnosis (all types except thyroid), aged 18 years or above, who agreed to participate in the study, irrespective of their disease or treatment continuum, and could read, write and speak one of the three target languages (Tamil, Telugu or Hindi) were included in this study. HNC patients considered medically or neurologically inept by treating medical professionals or those with psychiatric comorbidities (assessed by medical professionals as having psychotic manifestations or on psychiatric medications) were excluded. Psychotic manifestations are characterized by thoughts, feelings and behaviour evidencing a varying degree of personality disintegration and distortion of reality in various spheres [26, 27]. These were assessed by medical professional.

### Sampling method

We devised a purposive sampling method, to recruit HNC patients (excluding thyroid cancer), meeting the eligibility criteria from study sites. The sampling method focused on reflecting the range and diversity of the sample, including characteristics such as age, gender, educational qualification, HNC type, disease stage, treatment type and status, and substance use (tobacco and alcohol).

A minimum of 200 eligible HNC patients speaking each target language were recruited for the study. The sample size was determined following the rule of thumb sample size calculation for quantitative studies, which recommends ten participants per item in a questionnaire [22, 28].

### Recruitment and data collection

Oncologists, psycho-oncologists, nurses, and clinic or ward assistants at participating hospitals were asked to identify HNC patients meeting the inclusion criteria and refer them to the researchers on-site.

Referred HNC patients were approached by researchers (CS or NA) who explained the purpose of the study and what would be involved if they participated. CS was a female PhD candidate with previous experience in research and a registered clinical psychologist; NA was a female research assistant with a post-graduation qualification in Psychology and previous experience in qualitative and quantitative research.

Patients who expressed interest in the study were provided with a participant information sheet that described the research project in detail and the risks, benefits and costs involved in participating. Participants had the opportunity to ask any questions before being asked to sign a consent form. Participants who gave written consent were provided with a hard copy questionnaire booklet consisting of demographic and clinical questions and the SAS and PHQ-9 PROMs in the target language. They were instructed to self-complete the questionnaires and return them as soon as they completed it. Participation in this study was voluntary and any participants were free to change their mind and withdraw consent at any point up to and during data collection without any reason or consequence. Returned booklets were checked for completion by the researcher and data were entered manually into REDCap database [29]. NA and CS entered the respective data they collected and manually cross-checked each other’s data entry against the original source for errors to ensure data accuracy.

#### PROMs

##### Zung’s self-rating anxiety scale

SAS is an anxiety measure assessing affective and somatic symptoms of anxiety with 20 items [30, 31]. SAS has 15 positively worded items and five negatively worded items (items 5, 9, 13, 17, and 19) assessed with a four-point scale from ‘none or a little of the time’ to ‘most or all of the time’. Total scores range from 0 to 80, with a clinical cut-off score of > 36 indicating ‘clinically significant anxiety’ in cancer populations [30–33]. SAS has evidence of good internal consistency reliability (Cronbach’s alpha of 0.82) and validity [30, 34, 35]. Exploratory factor analysis in a sample of college students showed four factors [34, 35]. The four factors were anxiety and panic (items 1,2,3,4 and 18), vestibular sensations (items 5, 9, 13, 17, and 19), somatic control (items 6,10,11,12 and 14) and gastrointestinal/muscular sensations (items 7, 8, 15, 16 and 20) [36].

##### Patient health questionnaire - 9

PHQ-9 is a self-report depression measure, consisting of nine-items and assesses depressive disorders, psychosocial stressors and functional impairment [21, 37] respectively. All PHQ-9 items are assessed with a four-point scale from ‘not at all’ to ‘nearly every day’. Total scores range from 0 to 27, with scores of *≥*5, *≥*10 and *≥* 15 representing mild, moderate and severe levels of depression for cancer populations [38, 39]. PHQ-9 has evidence for good internal consistency reliability (Cronbach’s alpha 0.89) and validity [37, 40, 41]. Factor analytic studies revealed both single factor and two-factor structure (one factor based on somatic items and another factor based on affect) [42, 43].

##### Health-related quality of life

The European Organisation for Research and Treatment of Cancer (EORTC) Quality of Life Questionnaire (QLQ-C30) is a self-report measure of cancer-specific HRQL. It consists of 30-items that assesses cancer-specific symptoms and functioning relevant to a broad range of cancer populations [44]. Items 1 to 28 are assessed with a four-point scale from ‘not at all’ to ‘very much’; items 29 and 30 are assessed with a seven-point scale from ‘very poor’ to ‘excellent’. Total raw scores are transformed to a 0–100 scale; high scores on functional scales indicate higher level of functioning and high scores on symptom scales/items indicate greater symptom burden [44]. The EORTC QLQ-C30 has been shown to be reliable (Cronbach’s alpha ≥0.70) and valid [44].

The EORTC QLQ Head and Neck 35 (EORTC QLQ-HN35) is a self-reported 35-item measure of HNC-specific symptoms [45]. Items 31 to 60 are assessed with a four-point scale from ‘not at all’ to ‘very much’; items 61 to 65 are assessed with a ‘yes’ or ‘no’ scale. Similar to the QLQ-C30, total raw scores are transformed to a 0–100 scale and high scores indicate greater symptom burden [45]. The EORTC QLQ-HN35 has been shown to be reliable (Cronbach’s alpha ≥0.70) and valid [45, 46].

### Statistical analyses

Analyses were performed using Statistical Package for the Social Sciences Version 25.0 (IBM Corp. Released 2017. IBM SPSS Statistics for Windows, Version 25.0. Armonk, NY: IBM Corp.).

#### Descriptive analyses

For all three languages, the demographic and clinical data of the sample were analysed using descriptive statistics.

#### Missing data and targeting

Data completeness was examined by analysing the percentage of missing data for all items and scales, and the percentage of computable scale scores (where minimum criterion was > 50%) [47]. If a participant did not answer a minimum of 50% of the items, then the scale score was not calculated and regarded as missing data. Floor and ceiling effects were studied by calculating the percentage of patients with the lowest and highest scores in PROMs assessed (where minimum criteria < 15%) [47]. Scale to sample targeting based on scale scores spanning across scale range was also determined.

#### Scaling assumptions

Scaling assumptions are useful to determine whether the item scores of a scale in a PROM can be summated. Scaling assumptions were evaluated by examining the item-total correlation (ITC). The ITC scores were corrected for overlap. The correlation between the item and the sum of all other items in the same scale were determined, where the minimum criterion for ITC was ≥0.30) [47].

#### Factor analysis

Exploratory factor analysis (EFA) using varimax rotated was conducted to assess the dimensionality of the measures. Eigenvalues greater than 1 and item factor loading greater than 0.3 were accepted [47, 48]. Suitability of the data for factor analysis was assessed using Kaiser-Myer-Olkin (KMO) measure of sampling adequacy (where criterion 0.6 was considered suitable) and the Bartlett test of Sphericity (where *P*-value of < 0.01 was considered suitable) [48].

Since both SAS and PHQ-9 have already been used in the literature, we used confirmatory factor analysis (CFA) to test the model formally and appraise the robustness of the relationship among the factors obtained in EFA. CFA was carried out using Lavaan and Structural equation modelling (SEM) Package in R Commander. The goodness-of-fit indices were examined without putting any limitations and adding new connections. As recommended by Hu and Bentler, an excellent fit was considered when Standardized root mean residual < 0.06 and, Comparative fit index ≥0.95 [49].

#### Reliability

Internal consistency reliability of each measure was assessed with Cronbach’s alpha coefficient of 0.7 or greater (considered acceptable for use at group level) [47, 49].

#### Convergent validity

To determine the convergent validity of the measures, Pearson’s correlation coefficient (> 0.3 considered satisfactory) was used. It was hypothesized that anxiety and depression constructs of SAS and PHQ-9 would be highly correlated with the emotional functioning scale of EORTC QLQ-C30. Criteria were used as a guide to the extent of correlations, as opposed to pass/fail benchmarks (high correlation *r* > 0.7; moderate correlation *r* = 0.3–0.7; low correlation < 0.3) [47, 49].

## Results

### Sample characteristics

A total of 633 participants were approached and obtained written consent from, by the researchers. However, only 621 returned completed questionnaire booklet. Of the 621 patients, 205 were Tamil, 216 Telugu and 200 Hindi speaking. The distribution of HNC patients by their demographic and clinical characteristics is presented in Table 1. Patients were aged between 20 and 82 years, with a mean age of 50 years. Of all participants, 81.5% were male, 86.3% were married, 61.7% were treated for oral cavity cancer, 42% were primary school graduates, and 19.8% were unemployed. Forty-three percent had stage III cancer, and 75% were undergoing treatment at the time of data collection.

**Table 1:** Sociodemographic and clinical characteristics for 621 patients across languages and in total

Socio demographics	Tamil (***n*** = 205)	Telugu (***n*** = 216)	Hindi (***n*** = 200)	Total (***n*** = 621)
Age: Mean, Median (Range)	52.83, 54 (19–82)	50.5, 49 (21–89)	45.98, 45 (21–75)	49.77, 49 (19–89)
Missing	1	3	0	4
Gender *n* (%)				
Male	163 (79.5)	170 (78.7)	173 (86.5)	506 (81.5)
Female	41 (20)	42 (19.4)	26 (13)	108 (17.5)
Missing	1 (0.5)	4 (1.9)	1 (0.5)	6 (1)
Marital Status *n* (%)				
Married/De facto	183 (89.2)	169 (78.2)	184 (92)	536 (86.3)
Divorced/Separated	3 (1.5)	7 (3.2)	1 (0.5)	11 (1.7)
Single	6 (2.9)	8 (3.7)	13 (6.5)	27 (4.4)
Widowed	11 (5.4)	27 (12.5)	2 (1)	40 (6.4)
Missing	2 (1)	5 (2.3)	0	7 (1.1)
Education qualification *n* (%)				
Primary level	60 (29.3)	127 (58.8)	74 (37)	261 (42)
Intermediate level	78 (38)	28 (13)	46 (23)	152 (24.5)
Higher secondary level	39 (19)	27 (12.5)	26 (13)	92 (14.8)
Diploma	10 (4.9)	21 (9.7)	0 (0)	31 (5)
University degree	14 (6.8)	10 (4.6)	44 (22)	68 (11)
Post graduate degree	1 (0.5)	3 (1.4)	9 (4.5)	13 (2.1)
Missing	3 (1.5)	0	1 (0.5)	4 (0.6)
Employment *n* (%)				
Employed	141 (68.7)	106 (49.07)	153 (76.5)	400 (64.4)
Unemployed	23 (11.2)	80 (37)	20 (10)	123 (19.8)
Homemaker	23 (11.2)	20 (9.2)	17 (8.5)	60 (9.7)
Training/education	1 (0.5)	2 (0.9)	4 (2)	7 (1.1)
Retired	1 (0.5)	1 (0.5)	5 (2.5)	7 (1.1)
Unable to work	13 (6.3)	1 (0.5)	0 (0)	14 (2.2)
Other	0	1 (0.5)	1 (0.5)	2 (0.3)
Missing	3 (1.5)	5 (2.3)	0	8 (1.3)
Type of Cancer *n* (%)				
Oral Cavity	94 (45.8)	108 (50)	181 (90.5)	383 (61.7)
Salivary Gland	2 (0.9)	6 (2.8)	1 (0.5)	9 (1.4)
Oropharynx	26 (12.6)	3 (1.4)	2 (1)	31 (5)
Larynx + Hypopharynx	35 (17.1)	9 (4.2)	0 (0)	44 (7.1)
Sinus gland	2 (0.9)	1 (0.5)	0 (0)	3 (0.5)
Tongue	35 (17.1)	35 (16.2)	9 (4.5)	79 (12.7)
Nasopharynx	9 (4.4)	7 (3.2)	0 (0)	16 (2.6)
Nasal Cavity	0	29 (13.4)	4 (2)	33 (5.3)
Unknown primary	1 (0.5)	11 (5.1)	0 (0)	12 (1.9)
Missing	1 (0.5)	7 (3.2)	3 (1.5)	11 (1.8)
Stage of disease *n* (%)				
I	3 (1.4)	8 (3.7)	14 (7)	25 (4)
II	34 (16.5)	24 (11.1)	63 (31.5)	121 (19.5)
III	114 (55.6)	69 (31.9)	84 (42)	267 (42.9)
IV	43 (20.9)	19 (8.8)	31 (15.5)	93 (14.9)
Missing	11 (5.3)	96 (44.4)	8 (4)	115 (18.5)
Treatment offered *n* (%)				
Radiation	205 (100)	177 (81.9)	199 (99.5)	581 (93.6)
Chemotherapy	198 (96.6)	121 (56)	33 (16.5)	352 (56.7)
Surgery	29 (14.1)	32 (14.8)	115 (57.5)	176 (28.3)
Treatment status *n* (%)				
Undergoing treatment	104 (50.7)	177 (81.9)	189 (94.5)	470 (75.7)
Survivor	97 (47.3)	39 (18.1)	11 (5.5)	150 (24.1)
Substance usage *n* (%)				
Yes	150 (73.2)	187 (86.5)	190 (95)	527 (84.8)
No	54 (26.3)	20 (9.2)	9 (4.5)	83 (13.3)
Missing	1 (0.5)	9 (4.1)	1 (0.5)	11 (1.7)
ECOG Performance Status *n* (%)				
0	51 (24.8)	25 (11.6)	99 (49.5)	175 (28.1)
1	110 (53.6)	75 (34.7)	77 (38.5)	262 (42.2)
2	23 (11.2)	71 (32.9)	17 (8.5)	111 (17.9)
3	16 (7.8)	26 (12)	5 (2.5)	47 (7.6)
4	4 (1.9)	13 (6.0)	1 (0.5)	18 (2.9)
Missing	1 (0.4)	6 (2.8)	1 (0.5)	8 (1.3)

### Missing data and targeting

The analyses of data completeness showed that the data quality was high, with computable scale scores ranging from 98.5% to 100%. Scale-to-sample targeting was good and scale scores spanned the scale range (i.e. not skewed and were within +/− 1 criteria) for all scales except SAS Tamil (1.046). Floor effects were within the 15% criterion for all scales (See Table 2).

**Table 2:** Scale level analyses – data completeness and targeting of all SAS and PHQ-9 across Tamil, Telugu and Hindi (*n* = 621)

Scales	Data completeness - Computable scale score (%)	Targeting						
		Possible score range^a^	Observed score range	Range mid-point	Mean score	SD	F/C effect (%)	Skewness
**Tamil**								
SAS	99.0	20–80	23–71	50	36.28	9.669	5.4/0/5	1.046
PHQ-9	98.5	0–27	0–23	13.5	6.55	5.139	12.4/0.5	0.768
**Telugu**								
SAS	100	20–80	20–56	50	35.34	7.732	0.5/0.5	0.228
PHQ-9	99.5	0–27	0–24	13.5	9.97	4.715	1.0/1.4	0.655
**Hindi**								
SAS	98.5	20–80	24–63	50	34.91	8.294	0.5/0.5	0.910
PHQ-9	98.5	0–27	0–22	13.5	7.46	5.627	5.1/0.5	0.676

*SD* Standard deviation, *F/C* Floor/ceiling – floor effect = % scoring 100 (greatest bother/impact), ceiling effect = % scoring 0 (least bother/impact), *SAS* Zung’s self-rating anxiety scale, *PHQ-9* Patient health questionnaire −9

^a^High scores indicate great bother/impact

### Factor analysis

#### EFA

KMO measure of sampling adequacy for SAS (Tamil = 0.899; Telugu = 0.810; Hindi = 0.876) and PHQ-9 (Tamil = 0.850; Telugu = 0.811; Hindi = 0.899) were well above the recommended value of 0.6.

The Bartlett’s test of sphericity was significant for SAS (Tamil = 2393.328; Telugu = 1365.843; Hindi = 2472.075) and PHQ-9 (Tamil = 708.185; Telugu = 437.388; Hindi = 927.673) with *P* = 0.000, lesser than the recommended value of 0.001. Correlation matrices were inspected and found that all correlation coefficients were above 0.3, thereby the EFA results could be considered.

EFA results revealed the presence of four components (with eigenvalue exceeding 1) for SAS explaining the cumulative variance of 66%, 51.96% and 66.53% for Tamil, Telugu and Hindi respectively. (Please see online supplementary document for supplementary tables 1 and 2 containing factor loading output with items in both measures SAS and PHQ-9).

For PHQ-9, results revealed the presence of two components (with eigenvalue exceeding 1) explaining the cumulative variance of 61.26%, 51.45% and 67.59% for Tamil, Telugu and Hindi respectively.

#### CFA

The CFA results confirmed a four-factor model for SAS and a two-factor model for PHQ-9 across all three language versions. The results of the goodness-of-fit indices are presented in Table 3.

**Table 3:** Goodness of fit indices of SAS and PHQ-9 obtained from confirmatory factor analysis

Scales	Chi-square (χ2)	Degrees of freedom (*df*)	*P-value*	Standardized root mean square residual (S-RMR)	Comparative fit index (CFI)	The goodness of Fit index (GFI < 0.95)
**Tamil**						
SAS	560.479	165	0.00	0.085	0.829	0.767
PHQ-9	62.822	26	0.00	0.056	0.947	0.938
**Telugu**						
SAS	336.560	146	0.00	0.077	0.838	0.861
PHQ-9	71.90	26	0.00	0.064	0.888	0.935
**Hindi**						
SAS	754.854	146	0.00	0.128	0.741	0.711
PHQ-9	74.945	26	0.00	0.047	0.947	0.917

*SAS* Zung’s self-rating anxiety scale, *PHQ-9* Patient health questionnaire −9

### Internal consistency reliability

The Cronbach’s alpha coefficients for SAS and PHQ-9 across all three language versions were within the satisfactory range (> 0.70) (see Table 4).

**Table 4:** Internal consistency reliability and ITC for SAS and PHQ-9 across Tamil, Telugu, Hindi

Scales	Cronbach’s Alpha	Corrected ITC
**Tamil**		
SAS	0.868	0.311–0.690
PHQ-9	0.860	0.450–0.676
**Telugu**		
SAS	0.803	0.227–0.634
PHQ-9	0.717	0.395–0.605
**Hindi**		
SAS	0.863	0.292–0.719
PHQ-9	0.890	0.419–0.796

*SAS* Zung’s self-rating anxiety scale, *PHQ-9* Patient health questionnaire −9, *ITC* Item total correlation

### Within scale validity

Scaling assumptions were mostly satisfied based on ITC ≥0.3 for PHQ-9 across all languages and Tamil language version of SAS (see Table 4).

### Convergent validity

Correlations between SAS, PHQ-9 and the hypothesized scale of EORTC QLQ-C30 Emotional functioning were consistent with the predicted moderate positive correlations (*r* > 0.30) across all three languages (See Table 5).

**Table 5:** Scale level analyses – convergent validity across Tamil, Telugu, Hindi

Scales	Convergent Validity
	EORTC QLQ-C30 Emotional functioning scale r^**1**^
**Tamil**	
SAS	0.679*
PHQ–9	0.604**
**Telugu**	
SAS	0.669**
PHQ–9	0.574**
**Hindi**	
SAS	0.630**
PHQ–9	0.593**

*r*^*1*^ Pearson correlation, *SAS* Zung’s self-rating anxiety scale, *PHQ-9* Patient health questionnaire − 9

*Correlation significant at *p* = 0.05

**Correlation significant at *p* = 0.01

## Discussion

This study evaluated the psychometric properties of the Indian language versions of the SAS and PHQ-9 measures in a sample of Indian HNC patients speaking Tamil, Telugu and Hindi, demonstrating that the Indian language versions, like the original, are valid and reliable measures of anxiety and depression and appropriate for use in clinical and research settings with HNC patients.

For SAS, our factor analyses results are comparable with the original measure’s four-factor structure [36]. Although a four-factor structure emerged in both our EFA and CFA of the Indian versions, only two out of four factors loaded with similar items. The two factors that emerged consistently with similar items in our Indian versions as well as the factor analytic study by Olatunji et al. were ‘anxiety and panic’ and ‘somatic control’. The remaining two factors ‘vestibular sensations’ and ‘gastrointestinal/muscular sensations’ displayed little congruence with only some items loading similarly. Items that loaded similarly in ‘gastrointestinal/muscular sensations’ across both studies were ‘body aches/pain’, ‘fatiguability’, ‘nausea/vomiting’ and ‘urinary frequency’. The item ‘nightmares’ loaded in ‘vestibular sensation’ along with items ‘dyspnea’, ‘insomnia’ and ‘sweating’ [36]. This discrepancy in findings could be because of three reasons. The first is that our study included a clinical sample unlike the original factor analytic study where the sample consisted of college students. Cancer populations are at risk for psychological morbidity and physical symptoms, unlike a sample of college students. SAS has items such as insomnia, urinary frequency, fatigue, palpitation, dizziness, body aches and pains that may also be side effects of cancer treatment and therefore the results from cancer patients are likely to differ from presumed healthy college students. The second reason may be attributed to social desirability, as some patients may be embarrassed and/or have difficulty admitting their concerns about anxiety when facing a terminal illness such as cancer. Finally, the placement of negatively worded questions in between positively worded ones may have been confusing in some way, therefore preventing participants from using the full possible range of response options available in the PROM. Since about 42% of our total sample had only primary level education, it is more likely that they found it confusing to answer negative questions. Despite inconsistency in which specific items loaded into the four factors, clinicians and researchers are more likely to use the SAS as a severity or outcome measure that assesses a single construct as it was originally designed to do, rather than four separate outcomes for anxiety and panic, somatic control, vestibular sensation and gastrointestinal/muscular sensation.

For PHQ-9, our factor analyses (EFA and CFA) findings were comparable with the original measure’s two-factor structure (one factor based on somatic items and another factor based on affect) [43, 50, 51]. Although other studies have found a single factor model to be a good fit, the two-factor model is a significantly better fit for this heterogenous HNC populations when examined with CFA [23, 38, 43, 51]. However, since PHQ-9 was originally developed to screen patients seeking treatment for mental health in primary care settings, a single score for depression as was originally proposed [21, 37] may have better clinical utility than two separate scores for somatic and affect.

Indian language versions of the SAS and PHQ-9 demonstrated good internal consistency reliability, comparable to findings for the original English versions [30, 41]. The internal consistency of the scales was confirmed by ITC scores, which were all above expected criteria except for Telugu and Hindi versions of the SAS. It is possible that correlations for the Telugu and Hindi versions of the SAS were impacted by the inclusion of several negatively worded items. According to linguistic theories, ambiguous or complex sentence structures overload the processing abilities of the reader [52, 53]. In general, respondents make sense of the structure of a sentence by looking into its components such as certain words [53, 54]. For instance, as soon as respondents see a word, they fit it into a structure of the sentence and interpret it and this is due to working memory limitations. So, in the case of ambiguous or negatively worded sentences, it leads to (i) errors in interpretations, (ii) incorrect responses and (iii) re-analysing sentences which may increase the cognitive effort required to process them [52–54]. This is supported by similar findings by Jegede, et al. [55] that found the negatively worded items of the SAS had the lowest ITC scores and did not discriminate well between factors. Almost all ITC scores for every single item (when deleted) were lower than the overall correlation. This indicated that deleting items in the measures would not contribute to an increase in the overall reliability. So, all items were retained to ensure there was equivalence between the Indian language versions and the original English version of SAS.

Convergent validity of the Indian versions of SAS and PHQ-9 were supported by moderate to strong correlations between the measure’s constructs and the emotional functioning scale of the EORTC QLQ-C30. This suggests that the Indian versions of the SAS and PHQ-9 measures assess anxiety and depression, respectively, as originally intended. These findings are comparable with other studies that demonstrated that the SAS significantly correlated with other measures of anxiety [36] and the PHQ-9 strongly correlated with other measures of depression [40, 51]. Future work is needed to evaluate discriminant validity, clinical validity (known groups) and responsiveness of the Indian versions of SAS and PHQ-9.

Although being frequently employed in research studies, since their initial development and validation, PHQ-9 and SAS have both been consistently used in primary care and medical specialty clinics to screen for depression and anxiety respectively [56]. Additionally, PHQ-9 was developed from the Primary Care Evaluation of Mental Disorders (PRIME-MD) interview, which was the first instrument designed for use in primary care to diagnose disorders based on the Diagnostic and Statistical manual of Mental Disorders - 5 (DSM – 5) criteria. Both SAS and PHQ-9 have been reasoned to have content reflecting the standard DSM criteria used for assessing anxiety and depression respectively [18]. However, further work may be needed to ensure clinical utility for use in clinical practice for individual decision making.

Psychological support for HNC patients is needed from the time of their first consultation, through their diagnosis, treatment, post-treatment phase and into long-term post-treatment survivorship to minimize the psychological impact of a debilitating HNC experience. According to previous studies, Indians with HNCs have certain sociodemographic characteristics such as low socioeconomic backgrounds, lower education, poor social network and high prevalence of risk factors such as tobacco (smokeless and smoking) and alcohol use [12, 57]. Since these factors may additionally impact mental health and HRQL, healthcare professionals should take these into consideration. Additionally, these are also important to identify barriers that patients face when accessing psychological support. Healthcare professionals should also consider conducting a culturally sensitive semi-structured interview in addition to administering screening measures. This is because, in general, Indian patients show social desirability, reluctance to reveal their concerns to their healthcare provider, and an inclination to minimize the severity of symptoms or consider psychological distress as a normal reaction to a cancer diagnosis. Nearly 50% of cancer patients are diagnosed with anxiety and depressive disorders. Since HNC populations are more at risk for psychological morbidity, all patients should be screened for anxiety and depression to enable detection and subsequent supportive care.

Some limitations should be considered when interpreting the results of this study. First, only one cancer centre per language group participated, indicating our results may not reflect the broader experiences of patients in a large, populous country like India. Second, only those participants who were literate or semi-literate were approached for the study, excluding those who were illiterate. While India has made significant progress in improving literacy, the number of individuals who are illiterate continues to exist and increase.

The findings may not generalise to all HNCs as the majority of participants were diagnosed with oral cavity cancer and underwent radiation. Future work could evaluate the psychometric properties of the Indian versions of the SAS and PHQ-9 in mixed HNC populations. However, the present analyses are the first to report the factor structure of the SAS and PHQ-9 in an Indian sample. The factor structure of the SAS could be examined in HNC populations across different cultures as previous studies have suggested that the structure of somatic symptoms may differ across cultures [36, 58].

Another consideration is that screening of psychological morbidities using PROMs may have limited application for populations with low literacy or lack of familiarity with completing such forms [59]. Since SAS and PHQ-9 can be used as both self-reported and clinician-reported measures, having translated and culturally adapted versions will help healthcare professionals to use them on patients.

## Conclusions

In summary, the Indian language versions of Zung’s Self-rating Anxiety Scale and the Patient Health Questionnaire-9 measures can be used to assess anxiety and depression in Indian HNC patients. These PROMs can be used in comparative effectiveness research, multinational clinical trials, and in clinical practice to provide healthcare professionals with easily identifiable areas of concern to enable appropriate supportive care.

## Supplementary Information

**Additional file 1: Supplementary Table 1.** Zung’s self-rating anxiety scale items only: Principal Component analysis (EFA) with Varimax rotation (Items with a factor-loading coefficient ≥ 0.3 are retained in each factor (scale)). **Supplementary Table 2.** Patient Health Questionnaire-9 items only: Principal Component analysis (PCA) with Varimax rotation (Items with a factor-loading coefficient ≥ 0.3 are retained in each factor (scale)).

## Data Availability

The datasets used and/or analyzed during the current study are available from the corresponding author on reasonable request. Participant information and hard copy questionnaires have been coded, de-identified and recorded in a study specific electronic database built using RedCap. This is a secure, password protected system that has been used across multiple research studies within the University of Sydney. Paper records are stored in the University of Sydney’s locked filing cabinets in a security-controlled area.
