# Supplementary material for: Patient-reported anxiety and depression measures for use in Indian head and neck cancer populations: a psychometric evaluation
Source: J Patient Rep Outcomes. 2021 Jun 7;5:44. doi: 10.1186/s41687-021-00316-y (PMC8184912; doi:10.1186/s41687-021-00316-y)
Supplement: Supplementary file 1 — Additional file 1: Supplementary Table 1. Zung’s self-rating anxiety scale items only: Principal Component analysis (EFA) with Varimax rotation (Items with a factor-loading coefficient ≥ 0.3 are retained in each factor (scale)). Supplementary Table 2. Patient Health Questionnaire-9 items only: Principal Component analysis (PCA) with Varimax rotation (Items with a factor-loading coefficient ≥ 0.3 are retained in each factor (scale)). [file 41687_2021_316_MOESM1_ESM.docx]

**Supplementary Table** **1** Zung’s self-rating anxiety scale items only: Principal Component analysis (EFA) with Varimax rotation (Items with a factor-loading coefficient ≥0.3 are retained in each factor (scale)).

| **Item** | **Factors and loadings (exploratory factor analysis)** | | | |
| --- | --- | --- | --- | --- |
|  | **Factor 1** | **Factor 2** | **Factor 3** | **Factor 4** |
| **Tamil** | | | | |
| SAS_1 | 0.883 |  |  |  |
| SAS_3 | 0.848 |  |  |  |
| SAS_2 | 0.832 |  |  |  |
| SAS_4 | 0.698 |  |  |  |
| SAS_18 | 0.627 |  |  |  |
| SAS_5 | 0.376 | 0.624 |  |  |
| SAS_9 |  | 0.617 |  | 0.327 |
| SAS_13 |  | 0.566 |  |  |
| SAS_17 |  | 0.800 |  |  |
| SAS_19 |  | 0.781 |  |  |
| SAS_6 |  |  | 0.777 |  |
| SAS_10 |  |  | 0.707 |  |
| SAS_11 |  |  | 0.707 |  |
| SAS_12 |  |  | 0.664 |  |
| SAS_14 |  |  | 0.525 |  |
| SAS_7 |  | 0.426 |  | 0.756 |
| SAS_8 |  |  |  | 0.740 |
| SAS_15 |  |  |  | 0.723 |
| SAS_16 |  |  |  | 0.715 |
| SAS_20 |  | 0.542 |  | 0.791 |
| **Telugu** | | | | |
| SAS_1 | 0.763 |  |  |  |
| SAS_3 | 0.734 |  |  |  |
| SAS_2 | 0.697 |  |  |  |
| SAS_4 | 0.610 |  |  |  |
| SAS_18 | 0.574 |  |  |  |
| SAS_5 | 0.533 |  |  |  |
| SAS_9 |  | 0.843 |  |  |
| SAS_13 |  | 0.804 |  |  |
| SAS_17 |  | 0.584 | 0.386 |  |
| SAS_19 |  | 0.477 |  |  |
| SAS_6 |  |  | 0.653 |  |
| SAS_10 |  |  | 0.633 |  |
| SAS_11 |  |  | 0.562 |  |
| SAS_12 |  |  | 0.526 |  |
| SAS_14 |  |  | 0.467 |  |
| SAS_7 |  |  |  | 0.678 |
| SAS_8 |  |  |  | 0.587 |
| SAS_15 |  |  |  | 0.549 |
| SAS_16 |  |  |  | 0.459 |
| SAS_20 |  | 0.329 |  |  |
| **Hindi** | | | | |
| SAS_1 | 0.851 |  |  |  |
| SAS_3 | 0.825 |  |  |  |
| SAS_2 | 0.764 |  |  |  |
| SAS_4 | 0.711 |  |  |  |
| SAS_18 | 0.702 |  |  | 0.331 |
| SAS_5 | 0.698 | 0.387 |  | 0.315 |
| SAS_9 |  | 0.641 |  |  |
| SAS_13 |  | 0.587 |  |  |
| SAS_17 |  | 0.314 |  | 0.323 |
| SAS_19 |  | 0.814 |  |  |
| SAS_6 |  |  | 0.807 |  |
| SAS_10 |  |  | 0.738 |  |
| SAS_11 |  |  | 0.701 |  |
| SAS_12 |  |  | 0.665 |  |
| SAS_14 |  | 0.348 | 0.598 |  |
| SAS_7 |  |  |  | 0.826 |
| SAS_8 |  |  |  | 0.769 |
| SAS_15 |  |  |  | 0.815 |
| SAS_16 |  |  |  | 0.656 |
| SAS_20 |  | 0.607 |  |  |

Factor 1 – Anxiety and panic

Factor 2 – Vestibular Sensations

Factor 3 – Somatic control

Factor 4 – Gastrointestinal/Muscular sensations

**Supplementary Table** **2** Patient Health Questionnaire-9 items only: Principal Component analysis (PCA) with Varimax rotation (Items with a factor-loading coefficient ≥0.3 are retained in each factor (scale))

| **Item** | **Factors and loadings (exploratory factor analysis)^a^** | |
| --- | --- | --- |
|  | **Factor 1** | **Factor 2** |
| **Tamil** | | |
| PHQ_2 | 0.804 |  |
| PHQ_4 | 0.793 |  |
| PHQ_1 | 0.744 |  |
| PHQ_9 | 0.742 |  |
| PHQ_6 | 0.619 |  |
| PHQ_3 | 0.617 |  |
| PHQ_8 |  | 0.877 |
| PHQ_7 |  | 0.845 |
| PHQ_5 |  | 0.641 |
| **Telugu** | | |
| PHQ_1 | 0.532 |  |
| PHQ_2 | 0.791 |  |
| PHQ_3 | 0.623 |  |
| PHQ_4 | 0.722 |  |
| PHQ_5 | 0.340 | 0.445 |
| PHQ_6 | 0.747 |  |
| PHQ_7 | 0.474 | 0.588 |
| PHQ_8 |  | 0.879 |
| PHQ_9 | 0.522 |  |
| **Hindi** | | |
| PHQ_2 | 0.828 |  |
| PHQ_8 |  | 0.779 |
| PHQ_6 | 0.778 |  |
| PHQ_5 | 0.767 | 0.447 |
| PHQ_3 | 0.754 |  |
| PHQ_4 | 0.723 |  |
| PHQ_7 |  | 0.651 |
| PHQ_1 | 0.916 |  |
| PHQ_9 | 0.718 | 0.331 |

Factor 1 – Somatic

Factor 2 – Affect
